# Biomaterial-Based Hemostasis: A Review of the Clinical and Functional Versatility of Oxidized Regenerated Cellulose

**DOI:** 10.7759/cureus.94602

**Published:** 2025-10-14

**Authors:** Deepa Kanagal, Karthik Rao, Peter Gathoga, Kabir Moharana, Rajas Patil, Purva Jaiswal, Venkataraman AP, Deepak TS

**Affiliations:** 1 Obstetrics and Gynecology, Father Muller Medical College, Mangalore, IND; 2 Department of Head, Neck Surgery and Oncology, Sri Shankara Cancer Hospital and Research Centre, Bengaluru, IND; 3 Department of Obstetrics and Gynecology, Jacaranda Maternity Hospital, Nairobi, KEN; 4 Biology, Stonehill International School, Bengaluru, IND; 5 Clinical Affairs, Healthium Medtech Limited, Bengaluru, IND

**Keywords:** adhesion prevention, oxidized regenerated cellulose (orc), postoperative bleeding control, surgical hemostasis, surgical site infection (ssi), topical hemostatic agent

## Abstract

Oxidized Regenerated Cellulose (ORC) is a widely used polysaccharide-based hemostatic agent known for its biocompatibility, absorbability, antibacterial properties, and ease of application. It has been employed across a broad range of surgical specialties as an adjunctive tool for controlling mild to moderate bleeding, reducing postoperative adhesions, and minimizing infection risks. This scoping review aimed to evaluate the clinical efficacy, safety, functional versatility, and future potential of ORC across diverse surgical applications, with a focus on its comparative performance against other topical hemostatic agents. A systematic search of PubMed Central and clinicaltrials.gov database was conducted, including filters for only clinical trials, controlled trials, and randomized controlled trials involving human subjects. A total of 31 studies (28 from PubMed Central and three from clinicaltrials.gov) met the inclusion criteria, encompassing various surgical disciplines. Data were extracted on clinical indication, comparator agents, outcomes, and adverse events.

ORC demonstrated consistent efficacy in controlling intraoperative bleeding, particularly in orthopedic, general, and gynecologic surgeries. It reduced total blood loss and postoperative drainage in specific contexts, though its hemostatic effect was less pronounced than fibrin-based sealants in high-pressure bleeding scenarios. It also contributed to adhesion prevention, particularly in pelvic and abdominal surgeries, and showed antibacterial activity against multidrug-resistant organisms. Adverse events were rare and typically unrelated to the material itself. In conclusion, ORC is a versatile, cost-effective hemostatic agent valued for its ease of use, rapid absorption, and intrinsic bactericidal properties. While newer, specialized agents may excel in specific high-risk scenarios, it remains a clinically indispensable option due to its balanced efficacy and safety profile, particularly in resource-limited settings. Further high-quality studies are warranted to solidify its evidence base across diverse surgical applications.

## Introduction and background

Topical hemostatic agents play an important role in modern surgical practice. The application of topical hemostatic agents should be considered as an adjunctive measure to primary hemostatic techniques. They are especially beneficial in cases where the efficacy of conventional methods, including suture ligation and electrocautery, is limited by tissue accessibility, fragility, or the diffuse nature of minor capillary bleeding, making their use impractical [1-3].

Among the array of hemostatic materials available, polysaccharide-based agents have gained prominence due to their biodegradability, abundance, biocompatibility, and minimal immunogenicity. These materials have evolved alongside clinical demands and technological advancements, expanding their use beyond hemostasis to include applications such as sealants, wound dressings, and drug delivery systems. One of the most widely adopted polysaccharide-derived hemostatic agents is Oxidized Regenerated Cellulose (ORC). ORC is a chemically modified form of cellulose, first developed in 1942 by Yackel et al. and introduced commercially in 1945 [4-7]. It is widely used due to its favorable clinical characteristics like biocompatibility, biodegradability, absorbability, low toxicity, ease of application, and bactericidal properties. Its versatility and efficacy have led to its integration across various surgical disciplines, including general surgery, neurosurgery, head and neck surgery, thoracic surgery, gynecology, and urology. However, certain contraindications must be considered to ensure safe use. These include the presence of active infection without adequate drainage, direct application to major vessels, encasement of nerves (especially in confined bony spaces), hypersensitivity to ORC, and intraoperative complications such as extensive fractures. Additionally, powdered forms should not be used in blood salvage circuits due to the risk of filter contamination. Caution is advised in patients with poorly controlled diabetes, immunosuppression, ongoing chemotherapy, coagulation disorders, severe obesity, and other comorbidities (e.g., renal or hepatic insufficiency) that may increase the risk of complications [6,8,9].

ORC is available in various physical forms, standard, knit, fibril, and non-woven, to suit different surgical needs and bleeding intensities [5]. It offers significant advantages, including low production cost, minimal risk of thrombotic complications, and a low potential for disease transmission. Additionally, it features a long shelf life, further enhancing its practicality for clinical use [5,8].

Despite its widespread use, emerging studies point to gaps in our understanding of ORC's full clinical potential, side-effect profile, and optimization in complex surgical environments. This scoping review aims to synthesize the current evidence from clinical trials on the efficacy, safety, and adjunctive benefits of ORC across various surgical disciplines [10].

ORC's mechanism of action

The hemostatic efficacy of ORC is driven by both chemical and mechanical actions, which work synergistically to promote rapid clot formation and wound stabilization. The position of the oxidation sites on the cellulose molecule determines the physicochemical properties of ORC, which in turn dictates its efficacy. Through a process of oxidation, hydroxyl groups on the cellulose backbone are converted into carboxyl groups, forming polyuronic acids [11]. This structural modification imparts both hemostatic and bactericidal properties [8]. The acidic environment created by ORC not only contributes to its biodegradability but also promotes vasoconstriction, denaturation of blood proteins, restriction of local blood flow, and red blood cell lysis, facilitating clot formation. It also inhibits bacterial growth, making it particularly useful in contaminated surgical fields [11,12].

Following its application over the site that needs hemostasis, ORC acts by two mechanisms. Firstly, it acts as a scaffold, forming a dense absorbent mass upon contact with blood. This temporary hemostatic scaffold provides a mechanical tamponade over the open capillaries and initiates clot development by activating the coagulation cascade [11]. ORC is often preferred over other hemostatic agents like gelatin foam due to its dual functionality in promoting clotting and inhibiting microbial growth [5]. Gelatin foam absorption occurs over weeks but is site-dependent, and is contraindicated in infected wounds because it may exacerbate the infection [13].

**Figure 1:**
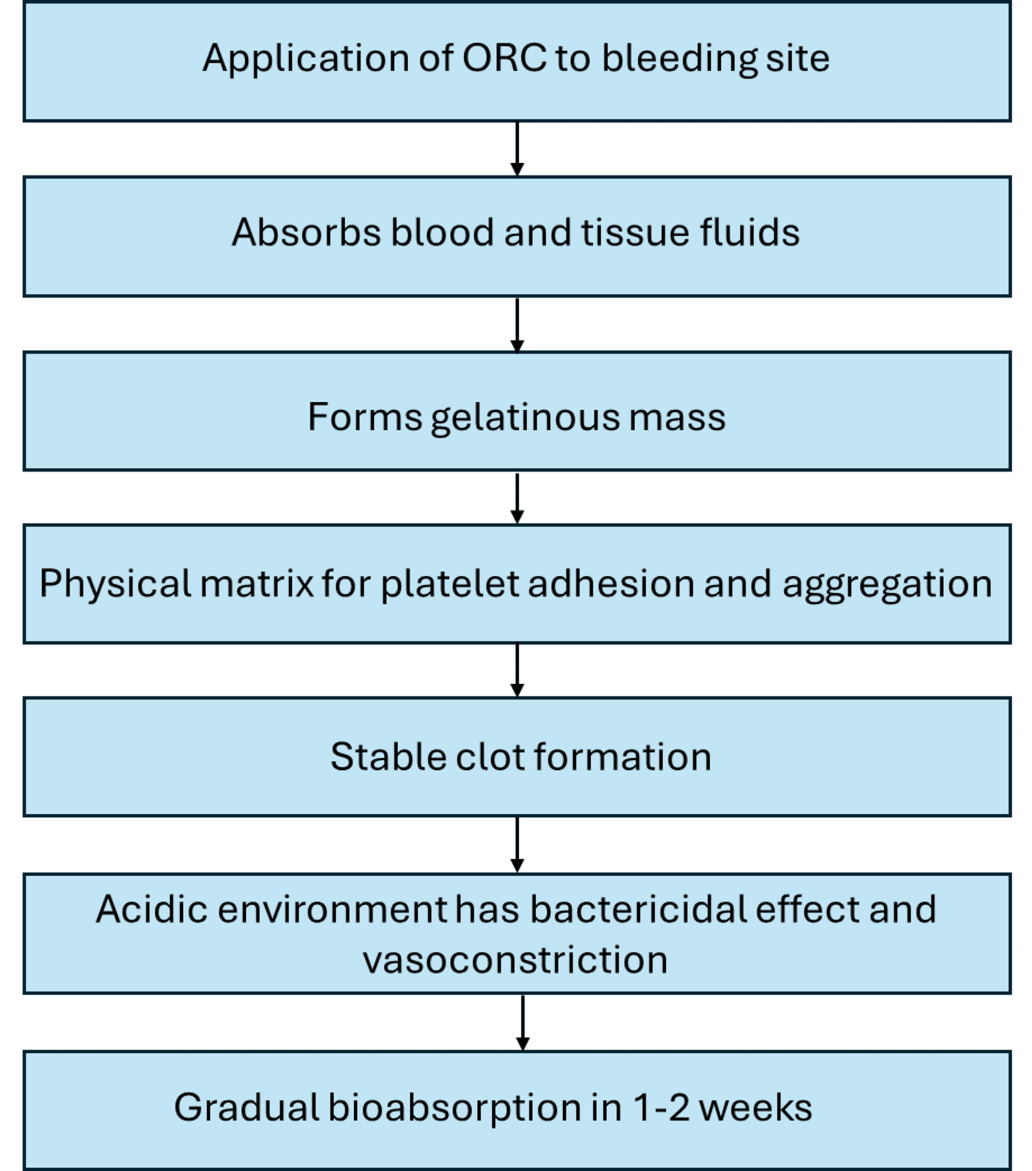
Mechanism of action of oxidized regenerated cellulose (ORC) Figure created by the authors on Microsoft Powerpoint (Microsoft Corp., Redmond, WA, US).

Secondly, the cohesive and adhesive structure of ORC enables better interaction with clotting factors, further supporting thrombus formation [14]. The large surface area of the cellulose fibers increases the absorption of blood and tissue exudates, thereby facilitating faster clot formation. More importantly, ORC is typically absorbed by the body within seven to 14 days (Figure 1) [1].

## Review

Methodology

A literature search was conducted using the PubMed Central database maintained by the U.S. National Institutes of Health (NIH). The search term “Oxidized Regenerated Cellulose” was used. The search results were filtered to include only the following types of studies: Clinical Trials, Controlled Clinical Trials, and Randomized Controlled Trials. Additionally, to explore the future clinical potential of ORC, a search was performed on clinicaltrials.gov database using the term "oxidized regenerated cellulose\ORC\". This yielded four relevant studies at various stages of completion. However, one of the completed studies was already identified in the PubMed search, resulting in three unique studies being included in the analysis. These were evaluated to identify prospective developments in the use of ORC across different surgical specialties and wound healing scenarios. The search was completed on June 26, 2025.

Studies were selected for inclusion based on specific criteria. Only studies conducted on human subjects and those reporting on the comparative efficacy or other clinically-relevant outcomes related to the use of ORC were included. Studies were excluded if they did not involve a direct comparison of ORC with another intervention or control group, assessed ORC in combination with other agents (as these could confound the interpretation of ORC’s standalone effects), retracted publications, and non-comparative studies.

Five independent reviewers screened all titles and abstracts for eligibility. Full texts of potentially relevant articles were assessed in detail. Any disagreements were resolved through discussion and consensus. From each eligible study, data were systematically extracted and organized according to the following categories: author(s) and year of publication, details of the intervention (specifically the use of ORC), comparator information (such as alternative hemostatic agents or control interventions), indication, reported outcomes, key results, and safety findings, including any adverse effects or complications.

As this is a scoping review, a formal risk of bias assessment was not performed. Due to the anticipated clinical and methodological heterogeneity of the included studies, a meta-analysis was not feasible. The results are therefore presented as a narrative synthesis.

This review explores several key domains related to the use of ORC. Firstly, it examines the comparative effectiveness of ORC in relation to other hemostatic agents or interventions, assessing its relative performance in clinical settings. Secondly, it discusses the side effects and complications associated with the use of ORC, highlighting potential risks and safety concerns. Lastly, it delves into ongoing research and future prospects, focusing on its advancements in clinical applications and material development.

Results

A comprehensive literature search in PubMed Central database yielded 725 records. After applying the filters, the number of articles were reduced to 54. These records were then screened for eligibility, based on the inclusion and exclusion criteria. A total of 26 studies were excluded for the following reasons: two did not involve ORC, one was a retracted article, five were not comparative in nature, and 18 used ORC in combination with another agent, making it difficult to isolate the effects of ORC.

A comprehensive literature search in clinicaltrials.gov database yielded four records. Since one of the completed studies was also retrieved in the PubMed search, only three non-duplicative studies were included in the analysis.

**Figure 2:**
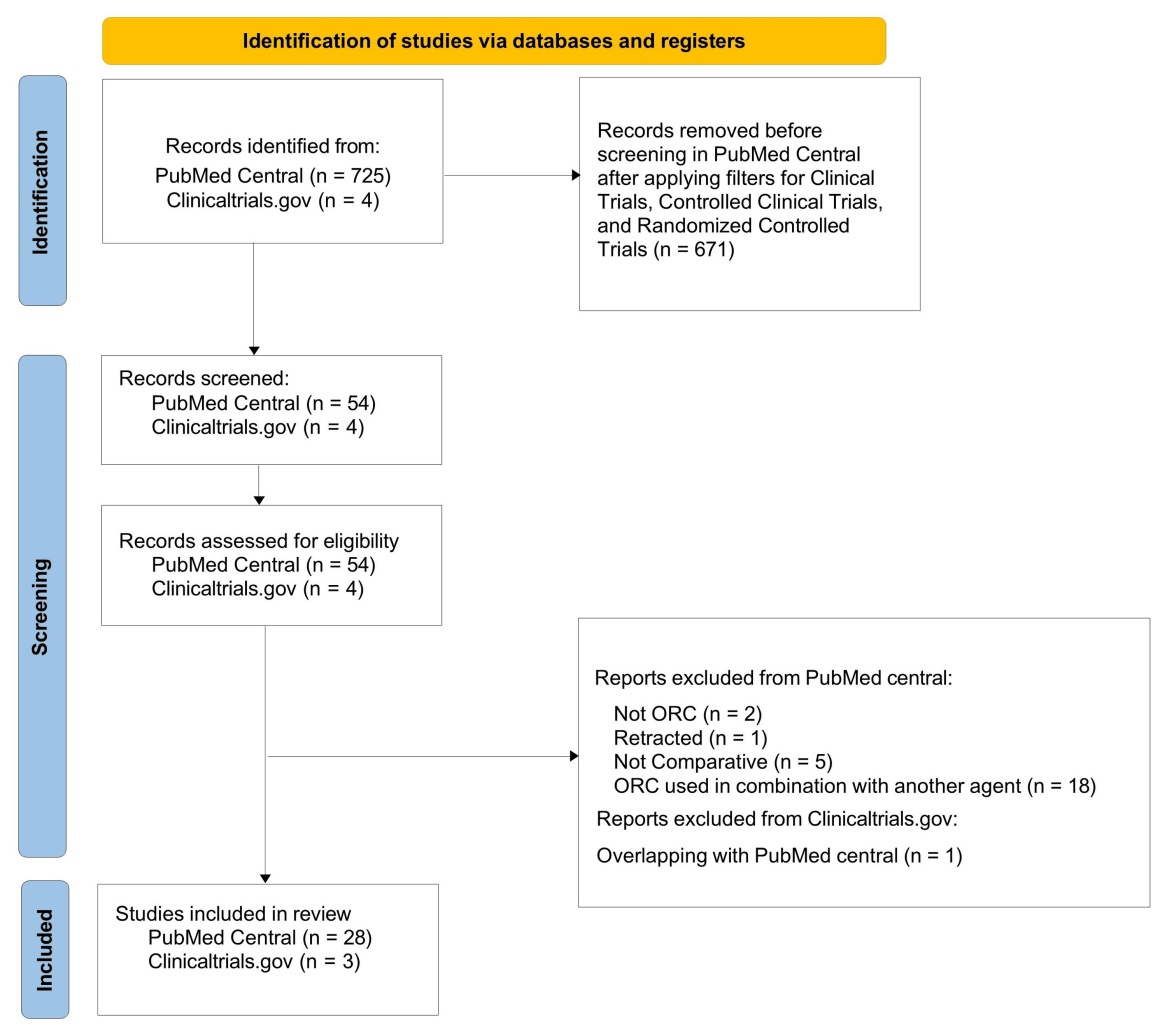
PRISMA Flowchart showing the literature search process The flowchart was prepared in accordance with the PRISMA 2020 (Preferred Reporting Items for Systematic Reviews and Meta-Analyses) guidelines [15].

Finally, 31 studies (28 studies from PubMed Central + three studies from Clinicaltrials.gov) were deemed suitable for the final review (Figure 2).

Across these studies, ORC was evaluated not only for its hemostatic properties but also for its effects on adhesion prevention, infection control, and postoperative recovery. A detailed summary of these 28 studies (from PubMed Central), including key findings and study characteristics, is presented in Table 1.

**Table 1:** Clinical studies evaluating ORC in surgical and medical settings ORC: Oxidized Regenerated Cellulose; PTFE: Polytetrafluoroethylene.

Sr No	Author & year	Intervention	Comparator	Clinical indication	Outcome	Results	Safety findings	Conclusion
Effect of ORC on blood loss								
1	Shimizu et al., 2019 [16]	ORC barrier (n=54)	Control (n=54)	Hemostasis at the video-assisted thoracoscopic surgery (VATS) access port site	Intraoperative blood loss	The median blood loss did not differ significantly between the ORC group [5 (0) mL] and the Non-ORC group [5 (3.75) mL] (P = 0.252)	No device related adverse effects	The use of an ORC sheet around a wound edge protector reduced interruptions from blood oozing, decreased the need for hemostatic procedures at wound closure, improved operative time, and enhanced the safety of VATS procedures
2	Li et al., 2023 [6]	ORC barrier (n=35)	Control (n=35)	Controlling post-operative bleeding after unilateral total knee arthroplasty (TKA)	Total, intraoperative, and hidden blood loss	Total and hidden blood loss were significantly lower in the ORC group compared to controls (902.32 vs. 1052.25 mL and 801.61 vs. 949.96 mL, respectively; P<0.05). Intraoperative blood loss was slightly lower but not significant (100.71 vs. 102.29 mL, P>0.05)	No device related adverse effects	ORC can effectively reduce postoperative blood loss
3	Wakasa et al., 2024 [17]	ORC powder (n=55)	Tranexamic acid (TXA) (n=56)	Controlling perioperative bleeding during total hip arthroplasty (THA)	Total and postoperative blood loss	There were no significant differences in estimated total blood loss between the ORC group (788.2 mL) and the TXA group (714.1 mL; p=0.141), nor in postoperative blood loss (437.5 mL vs. 332.1 mL; p=0.064)	No device related adverse effects	Topical administration of ORC powder is as effective as TXA in reducing perioperative bleeding in patients undergoing THA
Effect of ORC on drainage volume								
4	Nam et al., 2022 [18]	ORC + suction drainage (n=46)	Suction drainage alone (n=48)	Seroma prevention post-mastectomy	Comparison of total drainage volume	The mean total drainage volume was similar in patients treated with ORC + drainage (1134 ml) and those treated with drainage alone (1033 ml) (P=0.486)	No device related adverse effects	Use of ORC did not significantly alter the risk of seroma formation
5	Wang et al., 2015 [19]	ORC patch (n=20)	Control (n=20)	Reduce postoperative drainage volume after hepatectomy	Comparison of volume of drainage	The amount of drainage after operation was significantly reduced in the ORC group compared with the control group (406.9 vs 627.0 ml) (P<0.028)	No device related adverse effects	Application of ORC to the raw cut surface during hepatectomy significantly reduces postoperative drainage volume
Effect of ORC on the time to hemostasis								
6	Firmino et al., 2024 [20]	ORC dressing (n=15)	Calcium Alginate (CA) dressing (n=13)	Controlling bleeding from malignant wounds	Evaluation of total time to hemostasis (TTH)	The average TTH was similar in both the groups (67 sec vs 93.8 sec, P=0.894)	No device related adverse effects	Both ORC and CA are effective options for managing mild to moderate bleeding
7	Develle et al., 2020 [21]	ORC (n=118)	Neutralized ORC (NORC) (n=115)	Management of surgical wound bleeding during abdominal, thoracic, and vascular surgeries	Evaluation of total time to hemostasis and percentage of patients achieving hemostasis at 2, 5, and 10 minutes post-treatment	Hemostasis was significantly faster with NORC than ORC (median 36 sec vs. 67 sec; p<0.0001)	No device related adverse effects	Hemostasis was achieved more quickly in the NORC treatment group compared with the ORC group
8	Guardieiro et al., 2023 [22]	ORC gauze (n=60)	Chitosan based dental dressing (n=60)	Reduction of bleeding after dental extraction	Comparison of intra- oral bleeding time immediately after the dental extraction	Intra-oral bleeding time was significantly lower in chitosan dressing compared with ORC gauze (2 vs. 5 min, P=0.001)	No device related adverse effects	In patients undergoing dental extractions, chitosan dressing was superior to ORC gauze in reducing intraoral bleeding time within the first 20 minutes post-extraction, with both methods demonstrating a favorable safety profile
9	Rossman et al., 1999 [23]	ORC (n=9)	Absorbable gelatin sponge (n=9), Sterile gauze with external pressure (n=8)	Hemostasis management following palatal donor tissue harvesting for autogenous soft tissue grafting	Evaluate time to hemostasis in palatal donor wounds	The use of both the hemostatic agents significantly reduced median time to hemostasis in palatal wounds	Within the first 7 days post-surgery, bleeding occurred in 40% of both the ORC and control groups, whereas no bleeding events were reported in the absorbable gelatin sponge group	The use of hemostatic agents for palatal wounds is established as the preferred approach during free soft tissue graft procedures
10	Schenk et al., 2003 [24]	ORC (n=14)	Pooled human fibrinogen and thrombin (n=24), Manual pressure (n=10)	Achieve effective hemostasis during peripheral vascular surgery	TTH	The fibrinogen-thrombin group achieved significantly faster hemostasis, with a mean time of 56.3 seconds compared to 772.9 seconds in the ORC group and 1,269.6 seconds in the pressure group (P<0.0001)	No device related adverse effects	The fibrinogen-thrombin group achieved more rapid hemostasis compared to traditional techniques in peripheral vascular procedures, with no safety concerns observed
11	Schenk et al., 2002 [25]	ORC (n=2)	Fibrin sealant (FS) (n=10), Topical bovine thrombin (n=8), Bovine thrombin soaked cellulose Sponges (n=2), Pressure alone (n=6)	Achieving effective hemostasis after vascular anastomosis	TTH	The mean TTH was shortest in the FS group (29.3 s), followed by thrombin (147.4 s), thrombin-soaked cellulose sponge (346.0 s), pressure (872.2 s), and longest in the ORC group (1044.5 s)	No device related adverse effects	Fibrin sealant proved to be an effective topical hemostatic agent and demonstrated superiority over other treatment methods
12	Genyk et al., 2016 [26]	ORC gauze (n=110)	Fibrin sealant patch (FSP) (n=114)	Secondary treatment of local bleeding after hepatic resection	Hemostasis at the target bleeding site within 3, 5 and 10 minutes of application	Hemostasis within 3 min was achieved significantly more in patients in the FSP group than the ORC gauze group (80.7% vs. 50.0%, p<0.001)	Treatment-related adverse events occurred in 4.4% of FSP patients and 3.7% of ORC gauze patients. Serious events were reported in 2.6% of the FSP group (infectious peritonitis, liver abscess, postoperative adhesion) and 1.8% of the ORC gauze group (intra-abdominal fluid collection, peritoneal abscess)	FSP was well tolerated and demonstrated superior efficacy to ORC gauze as a secondary hemostatic treatment for operative sites in patients undergoing hepatic resection
13	Eshghi et al., 2014 [27]	ORC Tampon	Tranexamic acid impregnated tampon Chitosan reinforced tampon	Controlling epistaxis in patients with inherited bleeding disorders	Duration of hemostasis and the comparative efficacy in achieving hemostasis	The chitosan-reinforced tampon was significantly more effective in achieving rapid hemostasis (p<0.001)	No device related adverse effects	The chitosan-reinforced tampon was the most effective treatment for controlling epistaxis
Effect of ORC on adhesion prevention								
14	Mais et al.,1995 [28]	ORC barrier (n=25)	Control (n=25)	Prevention of adhesion formation following laparoscopic myomectomy	Adhesion-free rates were assessed by second-look laparoscopy at 12-14 weeks, with adhesion scores compared between groups	The ORC group had a significantly higher rate of adhesion-free patients (60% vs. 12%, p<0.05)	No device related adverse effects	The ORC absorbable barrier significantly reduced de novo adhesion formation following laparoscopic myomectomy
15	Haney et al., 1995 [29]	ORC barrier (n=32) (Each patient received PTFE on one side and ORC on the other)	Expanded PTFE barrier (n=32)	Prevent postsurgical adhesions after open reconstructive pelvic surgery	Adhesion score (on a 0–11 scale), adhesion area (cm²), and the probability of being adhesion-free	Both barriers reduced postoperative adhesion scores and area, but PTFE was significantly more effective, with a lower mean adhesion score (0.97 vs. 4.76) and smaller adhesion area (0.95 vs. 3.25 cm²) than ORC. More PTFE-covered sidewalls were adhesion-free (21 vs. 7), and even with contralateral adhesions, PTFE resulted in more adhesion-free sites (16 vs. 2)	No device related adverse effects	Expanded PTFE was associated with fewer postoperative adhesions to the pelvic sidewall compared to ORC
16	Mais et al., 1995 [30]	ORC barrier (n=16)	Control (n=16)	Prevention of adhesion reformation following laparoscopic treatment of endometriosis.	Reduction of adhesion reformation after laparoscopic endometriosis surgery, as evaluated by second-look laparoscopy	Adhesion-free outcomes were significantly more common in the ORC group (75%) than in the control group (12.5%) (p	No device related adverse effects	The ORC barrier significantly reduced adhesion reformation following laparoscopic surgery for endometriosis
17	Sawada et al., 2000 [31]	ORC adhesion barrier (n=23)	Control (n=15)	Prevent postoperative adhesions in infertile women undergoing reconstructive surgery	Comparison of postoperative adhesions	At second-look surgery, postoperative adhesions were observed in 37.5% (n=6) of the ORC group and 85.7% (n=6) of the control group. However, adhesion intensity and affected area did not differ significantly between groups	No device related adverse effects	The use of ORC significantly reduced postoperative adhesion formation and was associated with a statistically significant increase in pregnancy rates compared to surgical controls
18	Tinelli et al., 2011 [32]	ORC absorbable adhesion barrier (n=274)	Control (n=272)	Prevention of adhesion formation after intracapsular myomectomy	Presence of post-operative adhesions in four groups: laparotomy with barrier, laparotomy without barrier, laparoscopy with barrier, and laparoscopy without barrier	Adhesion rates were statistically highest in laparotomy without a barrier (28.1%), followed by laparoscopy without a barrier (22.6%), laparotomy with a barrier (22%), and lowest in laparoscopy with a barrier (15.9%)	No device related adverse effects	ORC as an adhesion barrier during intracapsular myomectomy significantly reduces postoperative adhesion formation
19	Franklin et al., 1995 [33]	ORC adhesion barrier (n=55) (one ovary was wrapped with ORC and the other was left uncovered)	Control (n=55)	Prevention of postsurgical ovarian adhesions in the treatment of bilateral ovarian disease	Ovarian adhesion presence and severity were assessed via second-look laparoscopy (10-98 days post-op), along with mean adhesion area reduction	At second-look laparoscopy, more ORC-treated ovaries were adhesion-free (26 vs. 14; p=0.028), with significantly greater improvement in adhesion severity (p=0.02). Mean reduction in adhesion area was greater with ORC (4.97 cm² vs. 3.08 cm²), approaching significance (p=0.055)	No device related adverse effects	Treatment of ovaries with ORC significantly reduced the occurrence and severity of postsurgical ovarian adhesions
20	Azziz et al., 1993 [34]	ORC barrier (n=134) (One pelvic sidewall covered by ORC and other left uncovered)	Control (n=134)	Prevention of pelvic sidewall adhesion reformation in patients undergoing adhesiolysis	Adhesion reformation incidence and adhesion area extent were assessed postoperatively	Adhesion re-formation was prevented in 68 of 134 sidewalls with ORC plus microsurgery, compared to 32 with microsurgery alone—more than doubling the success rate. ORC also significantly reduced the overall adhesion area	No device related adverse effects	The addition of the ORC barrier reduced the incidence, extent, and severity of postoperative adhesion re-formation
21	Sekiba et al., 1992 [35]	ORC barrier (n=63) (One pelvic sidewall covered by ORC and other left uncovered)	Control (n=63)	Prevention of postoperative adhesion reformation	Adhesion reformation incidence and adhesion area extent were assessed postoperatively	Significantly more adhesions were found on control pelvic sidewalls (76%) than ORC-treated sides (41%) during laparoscopy (p <0.0001)	No device related adverse effects	The ORC barrier effectively reduced both the incidence and extent of postoperative adhesions in patients with severe endometriosis
Other clinical outcomes with ORC								
22	Alfieri et al., 2011 [36]	ORC (n=50)	Iodine-soaked gauze (n=48)	Reducing the incidence of surgical site infections (SSIs) at previous stoma	Microbial contamination was assessed using 3 swabs: one intraoperatively before wound packing, and two on postoperative days 2 and 3 before skin closure	No or reduced bacterial contamination in second and third swabs was observed in 66% of ORC patients vs. 25% in the iodine gauze group	No device related adverse effects	ORC offers a clear benefit in reducing SSI risk, especially in patients with dirty surgical wounds
23	Naito et al., 2017 [37]	ORC (n=50)	Control (n=49)	Prevention of postoperative adhesions, particularly adhesive small bowel obstruction	Assessment of intraoperative ORC effectiveness, along with incidence of serious adverse events, SSIs, and adhesive bowel obstruction at 30 days and 6 months postoperatively	The overall adverse event rate was 12.0% in the ORC group and 16.3% in controls (p=0.58), showing no significant difference. ORC-related events included anastomotic leakage (3 cases), enteritis, intra-abdominal abscess, and strangulated ileus (1 case each), none attributed to ORC use. Adhesive bowel obstruction occurred only in controls (2 cases). ORC usability—assessed by incision length, delivery, trimming, site coverage, and adherence—was generally favorable	No device related adverse effects	ORC can be used safely and easily in laparoscopic colorectal surgeries
24	Scerrino et al., 2013 [38]	ORC gauze (n=60)	Collagen-Fibrinogen-Thrombin Patch (CFTP) (n=146) Standard thyroidectomy (n=65)	Acheive effective hemostasis after total thyroidectomy	Occurrence of post-operative complications	Seroma formation was significantly reduced in the CFTP group compared to ORC group (p=0.006) and the standard treatment group (p=0.017). No septic events were reported following CFTP application, while one hematoma occurred in the non-hemostatic group	No device related adverse effects	Both hemostatic agents reduced sero-hematic fluid accumulation within the first 24 hours post-surgery, with CFTP showing significant effectiveness than ORC gauze
25	Lee et al., 2017 [39]	Hemostasis-purposed ORC (n=20)	Adhesion barrier-purposed ORC (n=25)	As a filling material in the breasts after partial mastectomy	Weight of excised breast tissue, tumor size (clinical and pathologic), surgical margin status, operation time, hospital stay, and acute/chronic complications. Assessment of pathological factors and cosmetic outcomes over six months post-radiotherapy	Mean excised breast tissue weight was similar between groups (40.5 g vs. 42.9 g). The hemostasis-purposed ORC group had a significantly shorter operation time (65.3 vs. 90.6 min, p = 0.027) and lower post-radiotherapy SSI rate (p = 0.042). Tumor characteristics and treatment variables were comparable, and excellent cosmetic outcomes were observed in 55% and 56% of patients in the hemostasis and adhesion barrier groups, respectively	1 patient in the adhesion barrier-purposed ORC group developed a severe infection during radiotherapy and had poor cosmetic outcome.	The use of hemostasis-purposed ORC as a filling material for breast defects in breast cancer patients is superior to adhesion barrier-purposed ORC in terms of both surgical efficacy and cosmetic outcomes
26	Gatti et al., 2025 [40]	ORC (n=21)	Leukocyte‑and platelet‑rich fibrin (L‑PRF) membrane (n=21)	Treatment of palatal donor sites following free gingival graft (FGG) harvesting	Assessment of postoperative palatal donor site pain, discomfort, chewing difficulty, stress, surgical chair time, fibromucosa and graft thickness, and postsurgical complications within one week	Postoperative pain was similar between groups (p=0.326), while postoperative stress was significantly lower in the L-PRF group compared to the ORC group (p <0.05)	No device related adverse effects	Application of L-PRF membrane at palatal donor sites after FGG harvesting did not provide significant patient benefits
27	Testini et al., 2009 [41]	ORC patch (n=52)	Absorbable Collagen-Thrombin matrix (n=54), Hemostatic surgical procedure (n=49)	Adjunct to hemostasis when bleeding cannot be effectively controlled by ligature or conventional methods	Duration of operation, time to removal of wound drain, length of postoperative hospital stay, and incidence of postoperative morbidity	The Collagen-Thrombin matrix group had a significantly shorter mean operating time (105 min) than the surgical (133 min, p=0.02) and ORC groups (122 min, p=0.0003). Drain removal occurred earlier (p=0.006 vs. surgical; p=0.008 vs. ORC)	No device related adverse effects	Absorbable Collagen Thrombin matrix is an effective additional agent to conventional hemostatic procedures in thyroid surgery
28	Alkan et al., 2004 [42]	ORC (n=25) (One side served as control and on the other local hemostasis was achieved by ORC)	Control (n=25)	Achieve effective local hemostasis in patients experiencing facial swelling following the surgical removal of impacted mandibular third molars	Mean facial swelling values and reduction in mouth opening on postoperative days 1 and 3	There were no statistically significant differences between the test and control groups for the mean values of the facial swelling and mouth opening at day 1 and 3 post-operatively (P>0.05)	No device related adverse effects were identified	Establishing local hemostasis after the removal of impacted mandibular third molars is not highly effective in preventing postoperative facial swelling

Hemostatic Efficacy and Blood Loss Control

Several studies examined the ability of ORC to control blood loss in various surgical settings. In orthopedic surgery, Li et al. (2023) [6] reported a statistically significant reduction in both total and hidden blood loss with the use of ORC compared to the control group following unilateral total knee arthroplasty, highlighting the effectiveness of ORC in managing postoperative hemorrhage. Similarly, Wakasa et al. (2024) [17] showed that ORC powder was as effective as tranexamic acid in reducing blood loss during total hip arthroplasty, suggesting that ORC could serve as an alternative in patients where systemic antifibrinolytics are contraindicated. In thoracic surgery, Shimizu et al. (2019) [16] reported no statistically significant difference in median intraoperative blood loss between ORC-treated and control patients undergoing Video-Assisted Thoracoscopic Surgery (VATS), though ORC reduced the need for additional hemostatic interventions.

Impact on Drainage Volume

The use of ORC showed mixed results in reducing postoperative drainage. Wang et al. (2015) [19] demonstrated a significant reduction in drainage volume post-hepatectomy with the application of ORC compared to the control group (406.9 mL vs. 627.0 mL, p<0.028), suggesting its utility in liver surgery where fluid management is critical. However, other trials, such as that by Nam et al. (2022) [18] in mastectomy patients, did not find significant differences in seroma formation or drainage volume between the ORC and control groups, indicating that ORC’s benefits may be context-specific and dependent on surgical site characteristics and fluid dynamics.

Time to Hemostasis (TTH)

The ability of ORC to rapidly achieve hemostasis has been well documented, although its performance varies across indications and in comparison with other agents. In cases of low-grade bleeding, such as malignant wound oozing, ORC has shown comparable efficacy to other standard agents. Firmino et al. (2024) [20] reported that ORC and calcium alginate achieved similar TTH, indicating that ORC is effective in managing mild to moderate bleeding in oncology-related wound care. Conversely, in high-pressure bleeding scenarios such as vascular or hepatic surgeries, ORC was associated with longer TTH. Schenk et al. (2003) [24] demonstrated that fibrinogen-thrombin sealants achieved significantly faster hemostasis compared to ORC in vascular procedures (mean TTH: 56.3 vs. 772.9 seconds). Similarly, Schenk et al. (2002) [25] and Genyk et al. (2016) [26] found that fibrin sealant patches outperformed ORC gauze in hepatic resections and vascular anastomosis, providing faster and more reliable control of bleeding. However, modified forms such as neutralized ORC (NORC) appear to offer improved performance. Develle et al. (2020) [21] found NORC achieved faster hemostasis than conventional ORC, with 100% of patients achieving hemostasis within two minutes, highlighting the potential for material optimization. 

In the dental setting, Guardieiro et al. (2023) [22] compared ORC gauze with a chitosan-based dental dressing following tooth extraction. The chitosan group achieved significantly shorter intraoral bleeding times (two minutes vs. five minutes, P=0.001), suggesting that while ORC is effective, chitosan dressings may offer superior hemostatic control in oral surgical procedures.

Rossman et al. (1999) [23] evaluated hemostatic methods following palatal donor tissue harvesting. Both ORC and absorbable gelatin sponge significantly reduced the TTH compared to standard gauze pressure. However, postoperative bleeding occurred in 40% of both the ORC and gauze groups, while no bleeding was reported in the gelatin sponge group, indicating a superior safety and efficacy profile for the gelatin sponge.

In patients with inherited bleeding disorders, Eshghi et al. (2014) [27] reported that the chitosan-reinforced tampon achieved significantly faster hemostasis compared to the ORC tampon and the TXA-impregnated tampon (P<0.001). These findings demonstrate that ORC provides moderate efficacy in high-risk patients but is less effective than chitosan in managing epistaxis under coagulopathic conditions.

Adhesion Prevention and Reformation

Eight studies evaluated ORC’s ability to reduce postoperative adhesions. In laparoscopic myomectomy, studies by Mais et al. (1995) [28,30] and Sawad et al. (2000) [31] showed that patients receiving ORC had significantly higher adhesion-free rates compared to controls. Similarly, Franklin et al. (1995) [33] reported fewer and less severe ovarian adhesions with ORC treatment as compared to control. Azziz et al. (1993) [34] and Sekiba et al. (1992) [35] conducted paired-site studies in which one side of the pelvic cavity was treated with ORC while the other served as a control, showing significant reductions in both adhesion incidence and extent on the ORC-treated side. While some studies (e.g., Haney et al., 1995 [29]) found expanded polytetrafluoroethylene (PTFE) barriers to be superior in terms of reducing adhesion severity and surface area, ORC remained advantageous due to its absorbability, ease of use, and reduced risk of foreign body reactions. Tinelli et al. (2011) [32] further demonstrated that ORC significantly reduced adhesion rates compared to the control group in patients undergoing intracapsular myomectomy, with consistent benefits observed across both open and laparoscopic surgical approaches.

Other Clinical Outcomes

Alfieri et al. (2011) [36] demonstrated that ORC reduced microbial contamination in contaminated stoma sites more effectively than iodine-soaked gauze (66% vs. 25% showed no/reduced contamination). Similarly, Lee et al. (2017) [39] found lower surgical site infection (SSI) rates with hemostasis-purposed ORC versus adhesion-barrier-purposed ORC in post-mastectomy patients (p=0.042), along with shorter operative times (p=0.027).

In colorectal surgery, Naito et al. (2017) [37] found no increase in adverse events with ORC, and adhesive small bowel obstruction occurred only in the control group, suggesting a favorable safety and usability profile.

Functional outcomes such as seroma formation, recovery time, and hospital stay were variably impacted. In thyroidectomy, Scerrino et al. (2013) [38] observed fewer seromas with fibrin patches than with ORC. Testini et al. (2009) [41] also reported shorter operative and recovery times with collagen-thrombin matrix compared to ORC.

In oral and periodontal procedures, ORC was less effective than newer materials. Gatti et al. (2025) [40] found no significant difference in pain or healing between ORC and leukocyte- and platelet-rich fibrin (L-PRF), though L-PRF was associated with reduced postoperative stress (p<0.05). Alkan et al. (2004) [42] showed no benefit of ORC compared to the control in preventing postoperative swelling after third molar extraction, suggesting limited effectiveness of ORC in oral surgical procedures involving soft tissue edema.

Taken together, these clinical trials reflect a growing trend toward expanding the clinical roles of ORC beyond bleeding control, including applications in infection prevention, adhesion reduction, and wound healing optimization. With its broad-spectrum antibacterial activity, biocompatibility, resorbability, and evolving formulations (e.g., powders and gels), ORC remains a promising candidate for future research and therapeutic innovation in both surgical and nonsurgical domains.

Discussion

This review underscores the versatility and clinical reliability of ORC as a topical hemostatic agent across a wide range of surgical and bleeding scenarios. In a comprehensive review of topical hemostatic agents, ORC was noted to have a moderate hemostatic effect, but excellent handling characteristics. It did not adhere to instruments, conformed well to tissue surfaces, and was fully resorbed within weeks. These properties support its practical advantages in surgery, minimizing both intraoperative disruption and postoperative complications [11]. In procedures involving mild to moderate bleeding, such as malignant wound care in palliative settings or during dental extractions, ORC appears to provide satisfactory control [3]. Firmino et al. (2024) [20] found no significant difference in time to hemostasis between ORC and calcium alginate, suggesting that ORC remains a viable and cost-effective option for low-grade bleeding. In dental applications, Guardieiro et al. (2023) [22] showed that while chitosan dressings reduced bleeding time more rapidly, ORC still achieved satisfactory hemostasis within minutes, with no associated adverse effects, confirming its continued relevance in oral surgical practice. Moreover, studies such as Rossman et al. (1999) [23] have shown that ORC significantly improves intraoperative hemostasis compared to conventional gauze pressure, especially in procedures like palatal donor site management. Although gelatin sponges showed slightly better outcomes in preventing postoperative bleeding, ORC remains an effective, resorbable alternative with minimal complications. These findings further emphasize that ORC performs well in soft tissue surgeries where mechanical support and biocompatibility are critical.

While some studies in high-pressure bleeding environments, such as vascular or hepatic surgeries, report faster hemostasis with fibrin-based sealants [24,26], ORC still provides a safe and effective option, particularly in cases where biologic sealants are either contraindicated or cost-prohibitive. Importantly, ORC does not rely on patient coagulation factors or fibrin formation, making it particularly valuable in patients with coagulopathies or anticoagulant use. Develle et al. (2020) [21] demonstrated that neutralized ORC achieved faster and more consistent hemostasis than conventional ORC, with all patients reaching bleeding control within two minutes. This highlights the potential of modified ORC formulations to further optimize outcomes while retaining its core advantages. While other agents may offer advantages in select high-risk scenarios, the continued refinement of ORC, such as the development of neutralized or composite forms, positions it strongly for expanded clinical use.

In addition to the hemostatic properties, ORC also has demonstrated the ability to inhibit the growth of 32 different bacterial strains, including multi-drug resistant organisms such as methicillin-resistant Staphylococcus aureus (MRSA), vancomycin-resistant Enterococcus (VRE), penicillin-resistant Streptococcus pneumoniae (PRSP), and Candida albicans, with inhibition rates ranging from 50-100%. The complete inhibition of PRSP, a major cause of pneumonia and meningitis, is especially noteworthy and highlights ORC’s potential as a preventive barrier against SSIs. In vitro studies further support its activity against both Gram-positive and Gram-negative pathogens, which may reduce postoperative infection rates [5,43].

In addition to its hemostatic and antimicrobial benefits, ORC has emerged as an effective adhesion barrier in abdominal and pelvic surgeries. Several studies [44-46] report its efficacy in preventing postoperative adhesions when applied in sheet form to surgical sites. A meta-analysis [47] found that ORC significantly reduced adhesion incidence, with no reported cases of reoperation for adhesive small bowel obstruction. Mechanistically, ORC converts into a gel within 24 hours and is phagocytosed by macrophages. It supports tissue repair by promoting fibroblast, epithelial, and endothelial cell activity-further enhancing surgical healing.

Ongoing and Future Research

Four clinical studies involving ORC are currently registered on clinicaltrials.gov. As one of the completed studies overlapped with the PubMed search results, only three distinct studies were included in the final analysis. The first trial (Kuo et al. [48]) is a prospective comparison evaluating ORC versus hyaluronic acid in thyroid and parathyroid surgeries. The study focuses on postoperative adhesion formation and patient-reported swallowing difficulties, measured over one year. This research highlights ORC’s potential dual function as both a hemostatic and an anti-adhesion agent-extending its applicability beyond bleeding control to enhancing postoperative recovery.

A second completed trial (Al-Attar et al. [49]) assessed the efficacy and safety of a powdered ORC formulation in managing mild to moderate intraoperative bleeding across various surgical specialties, including general, gynecological, urological, and cardiothoracic procedures. Hemostasis success rates at three, five, and 10 minutes were key endpoints, alongside postoperative complications such as thromboembolic events and rebleeding. The trial's broad scope and multicenter design underscore ORC’s versatility and favorable safety profile across diverse clinical contexts.

The third study (Stacey [50]), although primarily focused on topical growth factors and protease inhibitors in chronic wound healing, indirectly informs future directions for ORC use. While ORC was not the main intervention, the study reflects an evolving therapeutic interest in bioactive wound care solutions. This aligns with the emerging role of ORC-based materials with antimicrobial or regenerative enhancements, particularly in treating complex wounds like diabetic foot ulcers and venous leg ulcers. 

ORC consistently demonstrated a favorable safety profile, with no significant reported adverse events. Even in high-risk surgical settings (e.g., hepatic resection, vascular surgery, colorectal procedures, neck dissections, and skull base repairs), ORC use was not associated with increased infection, allergic reaction, or delayed wound healing. When adverse outcomes did occur, such as infections, seromas, or hematomas, they were either attributed to surgical technique or patient factors, not to specific to ORC alone. While newer materials and sealants may offer incremental improvements in specific clinical scenarios, ORC continues to stand out for its unique combination of hemostatic efficacy, ease of use, absorbability, broad antimicrobial action, and additional benefits such as adhesion prevention in GI surgeries and promotion of wound healing.

Limitations

While this review provides a comprehensive assessment of ORC across diverse surgical applications, several limitations warrant consideration. The studies included in this review vary widely in terms of surgical specialty, patient populations, endpoints, and comparators. This heterogeneity limits the ability to draw direct comparisons or conduct meaningful meta-analyses. For example, TTH and drainage volume were measured using different methodologies across studies, reducing consistency in outcome interpretation. Although several RCTs were identified, many studies were small-scale, retrospective, or lacked blinding. This introduces the potential for bias in outcome reporting and underlines the need for larger, rigorously designed clinical trials to confirm the efficacy of ORC, particularly in comparison with newer or more specialized hemostatic agents. Many included studies used a broad range of alternative materials, such as gelatin sponges, fibrin sealants, or chitosan dressings, without consistent controls. This diversity complicates the evaluation of ORC’s relative performance and cost-effectiveness across clinical contexts. Evidence suggests that ORC’s benefits are highly dependent on the surgical setting and type of bleeding. Few studies assessed long-term postoperative outcomes, such as adhesion reformation, chronic infection risk, or delayed wound healing, over extended follow-up periods. Furthermore, real-world data on cost-effectiveness, surgeon preference, and logistical considerations (e.g., ease of storage, waste) are insufficiently addressed in the current literature. While ORC has shown safety in standard adult populations, limited data exist on its use in pediatric patients, individuals with complex coagulopathies, or those undergoing repeat surgeries. These populations require further investigation to validate ORC’s safety and efficacy.

## Conclusions

ORC has established itself as a clinically valuable and functionally adaptable hemostatic agent across a wide spectrum of surgical disciplines. Its consistent performance in controlling low to moderate bleeding, along with its ease of application, biocompatibility, and rapid absorbability, make it a dependable option in routine and complex surgical procedures. Moreover, ORC’s intrinsic bactericidal properties and low immunogenicity extend its utility beyond hemostasis, particularly in contaminated surgical fields and infection-prone environments.

While certain advanced agents, such as fibrin sealants, chitosan-based dressings, and synthetic adhesion barriers, have demonstrated superior outcomes in specific high-risk or specialized procedures, they often come with higher costs or increased technical demands. ORC, by contrast, offers a balanced profile of efficacy, safety, and cost-effectiveness, which supports its widespread clinical adoption, especially in resource-constrained settings. Nevertheless, additional high-quality studies are essential to enhance the evidence base and enable clinicians to make informed, evidence-based decisions across diverse surgical contexts.
